# A trouble shared is a trouble halved: The role of family identification and identification with humankind in well‐being during the COVID‐19 pandemic

**DOI:** 10.1111/bjso.12470

**Published:** 2021-06-16

**Authors:** Svenja B. Frenzel, Nina M. Junker, Lorenzo Avanzi, Aidos Bolatov, S. Alexander Haslam, Jan A. Häusser, Ronit Kark, Ines Meyer, Andreas Mojzisch, Lucas Monzani, Stephen Reicher, Adil Samekin, Valerie A. Schury, Niklas K. Steffens, Liliya Sultanova, Dina Van Dijk, Llewellyn E. van Zyl, Rolf Van Dick

**Affiliations:** ^1^ Department of Social Psychology Goethe University Frankfurt Germany; ^2^ Department of Psychology and Cognitive Science University of Trento Italy; ^3^ Department of Biochemistry Astana Medical University Nur‐Sultan Kazakhstan; ^4^ School of Psychology University of Queensland Brisbane Australia; ^5^ Department of Social Psychology Justus‐Liebig‐University Giessen Germany; ^6^ Department of Psychology Bar‐Ilan University Ramat Gan Israel; ^7^ School of Business University of Exeter UK; ^8^ School of Management Studies University of Cape Town South Africa; ^9^ Department of Psychology University Hildesheim Germany; ^10^ Ivey Business School University of Western Ontario London Canada; ^11^ School of Psychology and Neuroscience University of St Andrews UK; ^12^ Department of Psychology of Religion and Pedagogy International Islamic Academy of Uzbekistan Tashkent Uzbekistan; ^13^ Department of Psychology Branch of Moscow State University Named for M.V. Lomonosov in Tashkent Uzbekistan; ^14^ Department of Health Systems Management Ben‐Gurion University of the Negev Beersheba Israel; ^15^ Human Performance Management Eindhoven University of Technology The Netherlands; ^16^ Optentia Research Focus Area, North‐West University Vanderbijlpark South Africa; ^17^ Department of HRM University of Twente Enschede The Netherlands

**Keywords:** health‐related anxiety, COVID‐19, family identification, identification with humankind, social identity approach, mental and physical health

## Abstract

The COVID‐19 pandemic has triggered health‐related anxiety in ways that undermine peoples’ mental and physical health. Contextual factors such as living in a high‐risk area might further increase the risk of health deterioration. Based on the Social Identity Approach, we argue that social identities can not only be local that are characterized by social interactions, but also be global that are characterized by a symbolic sense of togetherness and that both of these can be a basis for health. In line with these ideas, we tested how identification with one’s family and with humankind relates to stress and physical symptoms while experiencing health‐related anxiety and being exposed to contextual risk factors. We tested our assumptions in a representative sample (*N* = 974) two‐wave survey study with a 4‐week time lag. The results show that anxiety at Time 1 was positively related to stress and physical symptoms at Time 2. Feeling exposed to risk factors related to lower physical health, but was unrelated to stress. Family identification and identification with humankind were both negatively associated with subsequent stress and family identification was negatively associated with subsequent physical symptoms. These findings suggest that for social identities to be beneficial for mental health, they can be embodied as well as symbolic.

## Background

The World Health Organization declared the COVID‐19 outbreak in December 2019 in Wuhan, China, as a worldwide pandemic (Sohrabi et al., 2020). This international health crisis poses a common threat to humankind and has a tremendous impact on society, the economy and the environment (Chakraborty & Maity, 2020). Up to date (16 May 2021), over 160 million confirmed COVID‐19 cases and over 3.3 million confirmed COVID‐19‐related deaths were reported worldwide (World Health Organization, 2021). In Germany, where the present study was conducted, about 3.5 million people have been infected and, so far, over 86,000 deaths have been reported (Robert Koch Institute, 2021). The COVID‐19 pandemic is a (life) threatening and unpredictable stressor, which greatly impacts individuals’ mental and physical health as reports of significant increases in anxiety, stress, and depression have demonstrated (Wang et al., 2020a, 2020b; Wang, Xia, et al., 2020). In addition, as people are affected by the unfolding pandemic, they may experience a critical increase in anxiety for their own lives and the lives of other people. Yet, not all individuals likely suffer to the same degree because of the pandemic as contextual risk factors such as living in a high‐risk area (with higher numbers of infections) or having a family member infected with the coronavirus might elicit stronger strain experiences and physical health complaints.

To slow down the spread of COVID‐19, the German government – as most other governments worldwide – implemented strict contact restrictions, reducing interpersonal contacts to a bare minimum (Bundesregierung, 2020a). Even though these social restrictions have been effective in preventing the spread of the virus (Qian & Jiang, 2020), they have also increased social isolation and loneliness, making people more vulnerable to mental and physical health conditions (Jetten, Reicher, Haslam, & Cruwys, 2020; Killgore, Cloonen, Taylor, & Dailey, 2020). These observations frame the conflicting nature of the COVID‐19 pandemic: On the one hand, in order to help each other in not catching the virus, people need to avoid direct social contact. On the other hand, people draw strength from feeling socially well‐integrated and supported – especially in stressful situations. The theory behind this *social cure phenomenon* is the Social Identity Approach (SIA) which states that identifying and being part of multiple social groups is health beneficial (Berkman & Glass, 2000; Haslam et al., 2008; Holt‐Lunstad, Smith, & Layton, 2010; Steffens, Haslam, Schuh, Jetten, & van Dick, 2017). Yet, group memberships are not only characterized by physical contact, they also entail a psychological component so that people identify with others and experience the health‐related benefits from their social groups even if they are physically separate from other group members (Khan, Garnett, Hult Khazaie, Liu, & Gil de Zúñiga, 2020).

On this basis, the present two‐wave study examines the associations between family identification and identification with humankind with stress and physical ill‐health symptoms during the COVID‐19 pandemic. We chose family and humankind as social groups, because different forms of social identification characterize them. With social restrictions in place, families are often the only social group with which individuals can regularly interact and receive direct social support. Thus, people who identify with their families may benefit from the feeling of being socially integrated and supported. By contrast, identifying as part of humankind implies a more symbolic feeling of ‘being in this pandemic together’ with many group members never meeting and interacting with each other (cf., Anderson, 1991; Khan et al., 2020). Thus, both forms of identification allow testing if social identification of a direct or symbolic nature is negatively related to stress and physical ill‐health symptoms.

**Figure 1:**
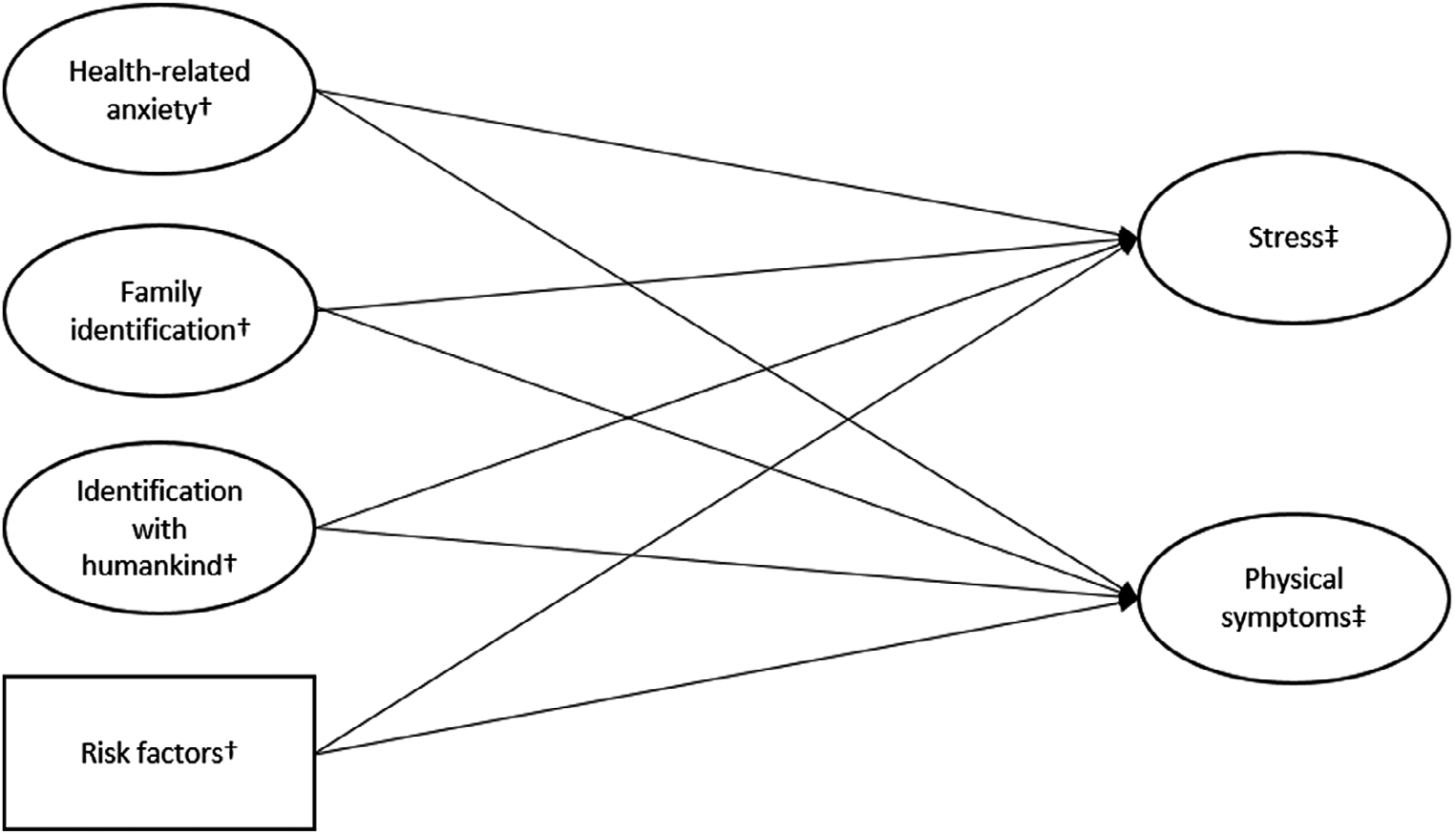
Proposed structural model and anticipated relationships between all variables. ^†^ measured at Time 1. ^‡^ measured at Time 2.

Therefore, we propose that health‐related anxiety and COVID‐19 risk factors are positively associated with stress and physical symptoms, whereas family identification and identification with humankind are negatively related to these two outcome variables (see Figure 1 for an overview of our proposed model).

### Health consequences of the COVID‐19 pandemic

Since the COVID‐19 outbreak, significant decreases in physical health and increases in self‐rated mental problems such as depression, distress, and anxiety have been reported in Germany (Bäuerle et al., 2020). In fact, experiencing any symptoms of COVID‐19, like a sore throat, headache, bodily pain or cough, was related to more stress, anxiety, and depressive symptoms, even when the affected individuals were not diagnosed with COVID‐19 (Rodríguez‐Rey, Garrido‐Hernansaiz, & Collado, 2020). In March 2020, when the current study was planned, the Robert Koch Institute (22 March 2020) classified the risk for the German population as ‘high’ and further emphasized that certain factors like age, medical preconditions, or the abode increase the risk of infection and of experiencing severe complications in case of infection. Thus, five COVID‐19 ‘risk factors’ were included in this study as they may negatively relate to mental and physical health. These are (1) caring for or (2) living with a person considered being at high risk, (3) having an infected family member or close friend, (4) living in or (5) having recently visited a high‐risk area. Although the COVID‐19 outbreak has been affecting people nationwide, these risk factors affect some individuals' health and well‐being more severely than others. With one or more of these factors applicable to an individual’s situation, they may see themselves and closely related others more exposed and vulnerable to the COVID‐19 pandemic and experience more mental and physical ill‐health. In the following, we will further emphasize these factors and their relations to mental and physical health conditions.

#### Consequences of the COVID‐19 risk factors for stress and health

The first two risk factors imply that people not only have to take care of their own mental and physical health, but also feel responsible for others who are at high risk (e.g., the elderly or people with chronic diseases) and/or whether they live close to such high‐risk people. Studies focusing on family caregiving showed health‐declining effects of caring responsibilities, whereby highly burdened caregivers reported the poorest health outcomes (Reinhard, Given, Petlick, & Bemis, 2008).

The third health‐threatening risk factor refers to the infection status of a closely related person. If a closely related person is infected with COVID‐19, individuals might be both concerned about their loved ones’ – and their own health status. Supporting the negative relation between overall health and infection status of a closely related person, results of a meta‐analysis on mental health consequences of the COVID‐19 pandemic indicate that suspected or confirmed COVID‐19 diagnoses of friends or family members were associated with more overall anxiety and depression (Vindegaard & Benros, 2020). Likewise, in a nationwide survey conducted in Italy, researchers found a positive association between COVID‐19 infection of a family member and overall anxiety and a relation between having an infected acquaintance and stress (Mazza et al., 2020). Furthermore, individuals with direct contact with COVID‐19‐infected individuals reported higher PTSD symptoms than people without close contact (Sun et al., 2021).

Living in or having recently visited a high‐risk area represent risk factors four and five. Even though the COVID‐19 pandemic ‘is more geographically and temporally diffuse than natural and human‐made disasters (Raker, Zacher, & Loew, 2020, pp. 12595), some regions have been more affected by the coronavirus than others (Sohrabi et al., 2020). We expected people who live in these so‐called high‐risk regions to experience more stress and physical symptoms because they have a higher vulnerability to be infected with the and thus might be more concerned about their health. In line with this assumption, residing in an area with many confirmed COVID‐19 cases has been associated with more psychological distress and stronger post‐traumatic stress responses in a Chinese sample (Sun et al., 2021; Wang, Xia, et al., 2020). The health‐declining effect of a person’s environment has also been found in the work context regarding different types of contagious diseases (e.g., SARS, AIDS, or hepatitis): Employees with a higher risk of infection due to their work environment (e.g., health care workers or correctional officers) reported more anxiety and job‐related stress (Chong et al., 2004; Hartley, Davila, Marquart, & Mullings, 2013). Further support for the environmental influence on health has been found in a non‐disease related context, where people reported more overall stress, anxiety, and more somatic symptoms due to their proximity to the Israeli and Lebanon border, a zone characterized by political uncertainty, war and terror at that time the study was conducted (Kimhi & Shamai, 2004).

Based on these results, we expect individuals to whom these COVID‐19 risk factors apply to experience higher stress perceptions and more physical symptoms. Taken together, we propose that:

H 1 Being exposed to more COVID‐19 risk factors at Time 1 positively relates to stress at Time 2.

H 2 Being exposed to more COVID‐19 risk factors at Time 1 positively relates to physical symptoms at Time 2.

#### The consequences of health‐related anxiety for stress and health

Besides the objective risk of infection, some people may be more anxious about becoming infected with the virus than others, affecting their stress and health symptoms. In line with this argument, Islam, Bodrud‐Doza, Khan, Haque, and Mamun (2020) showed that individuals concerned about family members’ health had an elevated stress response during the pandemic. Acute stress responses can be lifesaving by activating the fight‐or‐flight response, yet anxiety over a more extended period impairs the immune and cardiovascular system and increases people's vulnerability to become sick (Suinn, 2001). Thus, if a stressor like health‐related anxiety due to the COVID‐19 pandemic is active over a longer period, there might be a decline in psychological well‐being, particularly increased stress and a deterioration of one’s physical health. In line with this reasoning, studies demonstrated that living through a pandemic induces higher levels of health concerns (Li, Wang, Xue, Zhao, & Zhu, 2020) and general anxiety, as observed during past pandemics such as the swine flu in 2009 and the SARS‐1‐virus in 2003 (Chong et al., 2004; Wheaton, Abramowitz, Berman, Fabricant, & Olatunji, 2012). In fact, initial results on the relation between dysfunctional COVID‐19‐related anxiety, psychological distress, and physical ill‐health symptoms show that these are positively associated (Lee, 2020). On this basis, we propose that:

H 3 Health‐related anxiety at Time 1 positively relates to stress at Time 2.

H 4 Health‐related anxiety at Time 1 positively relates to physical symptoms at Time 2.

### Social identification and mental and physical health

Social identification, that is the feeling of being socially integrated and supported, is crucial for mental and physical health (Baumeister & Leary, 1995; Haslam, Jetten, Cruwys, Dingle, & Haslam, 2018; Steffens et al., 2017). The theoretical basis of this social identity‐health link is the *Social Identity Approach* (Haslam, 2004). As social beings, people tend to categorize themselves into different social groups and act according to the respective group's norms and values in group‐relevant situations. The individual’s social identity is the internalization of a group membership as part of the self.

Importantly, groups give us a sense of who we are and enable the individual to cope with stressful situations successfully. For instance, in an experimental setup, Haslam and Reicher (2006) assigned their participants to either the group of ‘guards’ or ‘prisoners’. The prisoners developed a shared social identity within their group, which buffered their stress responses, whereas the lack of a shared social identity within the group of guards lead to higher stress levels, even though they were – objectively – in a superior position. The prisoners’ subjective reports of perceiving less stress were further supported by reduced cortisol levels, emphasizing the negative association between social identification and physical health.

Group members who share a social identity provide more mutual social support (Haslam, Jetten, Postmes, & Haslam, 2009). Furthermore, perceptions of social support relate to greater collective self‐efficacy, which in turn relates to less strain (Avanzi, Schuh, Fraccaroli, & van Dick, 2015; Junker, van Dick, Avanzi, Häusser, & Mojzisch, 2019). However, to be health beneficial, social support has to be provided by people with whom the individual shares a social identity (Frisch, Häusser, van Dick, & Mojzisch, 2014).

Conversely, individuals who feel socially isolated and experience a reduction of their social contacts are more vulnerable to become ill (Cohen, Doyle, Turner, Alper, & Skoner, 2003), report more depression symptoms (Killgore et al., 2020), and have a higher mortality rate (Holt‐Lunstad et al., 2010). Indeed, results of a meta‐analysis indicate that the effects of loneliness and social isolation on life expectancy are as strong as the effects of other already well‐established health‐detrimental factors such as smoking (Holt‐Lunstadt et al., 2010). Therefore, social identification and the associated factors of social support (collective), self‐efficacy, sense‐making and providing meaning has also become known as a resource that can act as a ‘social cure’ (Haslam et al., 2018; Jetten, Haslam, & Haslam, 2012). In the following, we focus on two particular forms of social identification, namely family identification and identification with humankind and their relation to psychological and physiological health during the COVID‐19 pandemic.

#### Family identification and mental and physical health

Families can provide social support and easy access to social resources even in difficult situations like the COVID‐19 pandemic (Li et al., 2020). For example, family members provide emotional support by calming each other and providing a sense of security. Furthermore, families are an essential source of instrumental support, for instance, in the form of shopping for groceries for someone who does not feel safe leaving the house, sewing facemasks, or providing financial help. Due to the mutual support provided by family members’ collective self‐efficacy within the family increases, resulting in better overall health outcomes (see for a theoretical analysis: Häusser, Junker, & van Dick, 2020). Accordingly, and supporting this theoretical approach, family identification predicts better mental and physical health which can be attributed to the ‘we’‐feeling that arises from sharing a social identity (Bratt, 2015; Sani, Herrera, Wakefield, Boroch, & Gulyas, 2012; Wakefield, Sani, Herrera, Khan, & Dugard, 2016). Therefore, we propose that:

H 5 Family identification at Time 1 is negatively associated with stress at Time 2.

H 6 Family identification at Time 1 is negatively associated with physical symptoms at Time 2.

#### Identification with humankind and mental and physical health

Contrary to the family, the broader group ‘humanity’ is more symbolic and people cannot connect and mutually interact with everybody. Instead, contact is limited to interactions with only a few members of this group. Nevertheless, research shows that direct interactions with other group members do not necessarily enhance the benefits people derive from large‐scale group memberships (Khan et al., 2020). In fact, group contact frequency is not related to health and even just thinking about one’s social groups and thus making one’s own social identity salient has been associated with less depressive symptoms after dealing with stress and failure (Cruwys, South, Greenaway, & Haslam, 2015; Wakefield et al., 2016).

Even though humankind might be a less salient group than more proximal groups, such as one’s family, the COVID‐19 pandemic has been affecting individuals across the globe, which likely creates a shared identity and a feeling of ‘we are all in this together’ (Tajfel, Billig, Bundy, & Flament, 1971). This assumption is supported by the fact that people behave in favour of humanity and act according to group‐relevant norms and values, by staying at home for other people – not particularly for their own health protection, but to protect others (Jetten et al., 2020). One viral example of such collective caring behaviour is the *#IStayHomeChallenge* in the UK, where people posted statements of why and for whom they stayed home on their social media profiles (DeSantis, 2020, March 23). Focusing on the health of the collective does not only save lives, but the shared stressful experiences and mutual understanding also reduce stress and physical symptoms (Gallagher, Meaney, & Muldoon, 2014). Additionally, experiencing a collective trauma strengthens the identification within the affected community and enables a more adaptive reaction of the individual in the aftermath of the trauma (Muldoon, 2020; Muldoon et al., 2017). Therefore, we propose that:

H 7 Identification with humankind at Time 1 is negatively associated with stress at Time 2.

H 8 Identification with humankind at Time 1 is negatively associated with physical symptoms at Time 2.

## Method

### Participants and procedure

This study was conducted as part of a multi‐national research project on social identification during the pandemic and was approved by the Commerce Faculty Ethics Board (University of Cape Town; REF: REC 2020/03/013). For the present analyses, we used a sample from Germany. Participants in Germany were recruited via Kantar, an online panel provider and received 1.65 € for their participation per survey. Before completing the online questionnaire, participants had to agree with the informed consent statement. At the end of the first survey, the respondents were asked if they would like to participate in a follow‐up survey four weeks later. A total of 1484 individuals participated in the first survey (26 March – 31 March). We excluded nine cases because seven participants participated more than once. We used only responses of their first completions of the survey which resulted in a sample of *N* = 1475 at Time 1. Of these 1475, 1015 individuals also completed the second questionnaire four weeks later (27 April – 4 May). At Time 2, we identified five people who participated twice and only included their first responses leaving us with a sample of *N* = 1010 at Time 2 (attrition rate: 31.53%). Data collection started a few days after the German government implemented extensive contact restrictions (Bundesregierung, 2020a). The rapid changes of the pandemic progress and the associated political decisions made it difficult to plan an appropriate retest interval. On the one hand, a longer interval would have enabled us to focus on the longer‐lasting health consequences. On the other hand, a longer interval would also have increased the risk of external and uncontrollable factors (e.g., easing of restrictions) influencing our results. In the mid of April 2020 contact, restrictions were extended until 3 May 2020, but the first relaxation of restrictions was announced simultaneously (Bundesregierung, 2020b). Therefore, we used a 4‐week time lag to secure comparable circumstances at Time 1 and Time 2 as much as possible.

Buchanan and Scofield (2018) suggested that sufficient checks of data quality should consist of multiple indicators. Therefore, data quality was checked based on response patterns, response time, and answers given to an open answer format question. Participants were flagged when (1) their answer patterns across subsequent subscales did not differ (e.g., a constant response of *3 = ‘neutral’*), (2) their response time was less than 50% of the calculated median of the average response time of the sample (for a similar approach, see Kaluza, Weber, Van Dick, & Junker, 2021), or (3) their responses to an open answer format question was clearly insincere (e.g., typing random letters). The latter criterion was added because interpretable answers to open‐ended questions indicate participant motivation (Schmidt, Gummer, & Roßmann, 2020). Participants who got flagged two times were excluded from further analysis. Accordingly, at Time 1, 19 participants were excluded due to low data quality and 20 participants were excluded because they did not answer all five ‘risk factors’‐items. As we planned to calculate a weighted score based on all risk items, a missing value on one of the five items would entail a potential underestimation of the calculated risk. Thus, we decided to exclude cases with missing values. However, we also tested all hypotheses with these participants included into our analyses, which did not change our results. At Time 2, 19 participants were excluded due to questionable data quality. After matching both data sets, the final sample consisted of *N* = 974.

In the final sample, the majority of the participants (61.3%) were employed and 57.1% reported to have children. Only two participants (0.2%) had suffered from and one participant (0.1%) indicated to have been cured of COVID‐19. 40.3% of the participants had not been tested (*n* = 393) and 59.2% were tested negatively (*n* = 577).^1^

We compared the age (mean age 51.14 years, *SD* = 13.76) and gender distributions (52.7% women) of the final sample (*N* = 974) with the respective distributions of the German population obtained from the Federal Statistical Office (2020, June; 2020). The age distributions show strong resemblances; however, our sample comprised older people (especially in the age range from 40 to 59 years). Therefore, we decided to run all analyses with and without age included as a covariable to compare the results. The sample’s gender distribution shows a small surplus of women, which matches the German population's distribution (Federal Statistical Office, 2021, May). Additionally, participants were further asked to indicate their ZIP codes to compare the sample’s distribution across the federal states in Germany with that of the German population (see Table 1). Additionally, this allowed us to track COVID‐19 case numbers according to the participants’ current federal state of residence (see Table 2; most cases were recorded in Bavaria, Hamburg and Baden‐Wuerttemberg). Overall, the descriptive analysis indicates that the final sample is representative for the German population regarding age, gender, and federal state of living.

**Table 1:** Sample distribution across the German federal states based on *N* = 970

Federal states	Study Sample		Population in state of total population in Germany^†^	
	*N*	%	*N*	%
Baden‐Württemberg	117	12.1	11 070 000	13.3
Bavaria	150	15.5	13 077 000	15.8
Berlin	42	4.3	3 645 000	4.4
Brandenburg	30	3.1	2 512 000	3.0
Bremen	9	0.9	683 000	0.8
Hamburg	28	2.9	1 841 000	2.2
Hesse	73	7.5	6 266 000	7.5
Mecklenburg Western Pomerania	19	2.0	1 610 000	1.9
Lower Saxony	90	9.3	7 982 000	9.6
North Rhine‐Westphalia	191	19.7	17 933 000	21.6
Rhineland Palatinate	54	5.6	4 085 000	4.9
Saarland	11	1.1	991 000	1.2
Saxony	66	6.8	4 078 000	4.9
Saxony‐Anhalt	27	2.8	2 208 000	2.7
Schleswig Holstein	38	3.9	2 897 000	3.5
Thuringia	25	2.6	2 143 000	2.6
Total	970	100.0	83 021 000	100.0

Four participants did not indicate their ZIP code.

^†^ Federal Agency for Civic Education/bpb (2018, December 31).

**Table 2:** Categorization of participants’ living areas (amount and percentages of COVID‐19 cases per 100.000 population)

Cases per 100.000 population	
	*n* (%)
0 ‐ ≤ 5	0 (0)
> 5 ‐ ≤ 25	19 (2.0)
> 25 ‐ ≤ 50	195 (20.1)
> 50 ‐ ≤ 100	461 (47.53)
> 100 ‐ ≤ 150	295 (30.41) ^†^
> 150 ‐ ≤ 200	0 (0)
> 200 ‐ ≤ 250	0 (0)
> 250 ‐ ≤ 300	0 (0)
> 300	0 (0)
Total	970 (100)

Four participants did not indicate their ZIP code.

During the first survey period (March 26 – 31), the case numbers (per 100.000 population) were the highest in Hamburg, Baden‐Wuerttemberg, and Bavaria (Robert Koch Institute, 2020, March 31).

^†^ Bavaria (*n* = 150), Hamburg (*n* = 28), and Baden‐Wuerttemberg (*n* = 117) .

### Measures

Besides providing demographic information, participants completed the following items and scales^2^. Unless stated otherwise, all items were answered on a five‐point Likert scale from *1* = *strongly disagree* to *5* = *strongly agree,* and a scale mean was created for each measure. The complete scales are provided in the Appendix.

#### Health‐related anxiety (Time 1)

Health‐related anxiety was measured with five items, each referring to a different social group (sample item: ‘At the moment, because of the coronavirus pandemic, I am feeling anxious about my family/ close friends getting seriously ill.’). These items were based on the marker item for state anxiety (‘I feel anxious’) in the State‐Trait Anxiety Questionnaire (Spielberger, Gorsuch, Lushene, Vagg, & Jacobs, 1983) and adapted to refer to the COVID‐19 pandemic. Cronbach alpha was .85.

#### COVID‐19 risk factors (Time 1)

Building on the five risk factors described before, we asked participants to answer the following questions with *0* = *no* or *1* = *yes*: ‘Are you taking responsibility for people who are exposed to high risk (e.g., taking care of parents/grandparents)?’, ‘Are people who are exposed to high‐risk (elderly, chronically ill) living in your household or close by?’, ‘Has a family member or close relative/ friend been diagnosed with the coronavirus?’, ‘Do you live in a high‐risk area (with many documented cases of the coronavirus)?’, and ‘Have you recently visited a high‐risk area?’.

The degree to which these five risk factors contributed to an additional burden was rated by eleven health and social psychologist who all resided in Germany at the time of data collection. They were asked to compare the five items regarding their burdening degree with each other. Based on their individual perspectives, they assigned a respective percentage value to all five items and ensured that the sum is 100. Hence, when all factors applied to an individual, the burdening degree due to the risk factors would be 100%. Based on these ratings, a mean weight was assigned to the respective statement and an overall ‘risk score’ was calculated (mean weights can be found in the Appendix). For example, the raters assigned a mean weight of *M* = 9.55 (i.e., roughly 10%) out of 100 (100%) to the aspect ‘recently visited a high‐risk area’. In order to calculate a risk score ranging from 0‐1, the mean weights were divided by 100 and included into the equation. Higher values indicate a higher burden due to the COVID‐19 pandemic. The intraclass correlation coefficient (ICC) was .91 (*p *< .001), indicating a high inter‐rater reliability (Cicchetti, 1994; Koo & Li, 2016). Because only two participants were infected with COVID‐19, this item was not included in the final risk score calculation.

#### Family identification (Time 1)

Four items for measuring individual identification (Doosje, Ellemers, & Spears, 1995) were adapted to operationalize family identification (sample item: ‘I identify with my family’.). Cronbach alpha was .97.

#### Identification with humankind (Time 1)

In order to measure identification with humankind, the same four items by Doosje et al., (1995) were adapted (sample item: ‘I identify with other human beings’). Cronbach alpha was .90.

#### Stress (Time 2)

Stress was assessed with the short form of the Perceived Stress Questionnaire (Fliege, Rose, Arck, Levenstein, & Klapp, 2001). Participants were asked to indicate how often the following statements applied to them in the last four weeks on a scale from *1* = *almost never* to *4* = *usual*. The subscales ‘Worries’ (sample item: ‘You feel frustrated’), ‘Tension’ (sample item: ‘You feel tensed’), ‘Joy’ (sample item: ‘You feel you are doing things you really like’), and ‘Demands’ (sample item: ‘You have too many things to do’) can be calculated. As we were interested in the participants’ overall stress experience (for a similar approach, see Biehl, Boecking, Brueggemann, Grosse, & Mazurek, 2019), we collapsed all items into an overall stress score with a Cronbach alpha of .93.

#### Physical symptoms (Time 2)

Physical symptoms were measured with the brief form of the Giessen Subjective Complaints List (GBB‐8; Kliem et al., 2017). The participants were asked to indicate how often they experienced eight physical symptoms (sample item: ‘Stomach ache’) in the last four weeks on a scale from *1* = *never* to *6* = *very often*. Cronbach alpha was .87.

### Statistical analyses

A sequential data modelling strategy was adopted using SPSS 25 (IBM, 2017) and Mplus v. 8.3 (Muthén & Muthén, 2019). First, descriptive statistics (means, standard deviations, skewness, kurtosis) and Pearson correlation coefficients were calculated. Data were considered normally distributed if the skewness and kurtosis thresholds did not exceed a range of −2 and +2 (Field, 2009).

Second, a confirmatory factor analytical approach using structural equation modelling (SEM) was conducted to determine the best‐fitting model for the data. Here, five different confirmatory factor analytical (CFA) models were fitted to the data and systematically compared. Model fit was established by considering both model fit statistics as well as measurement quality (Shi & Maydeu‐Olivares, 2019). The maximum likelihood parameter estimation with robust standard errors (MLR) was used both for the measurement and structural models, as it provides more robust estimations in case of non‐normal data distribution (Muthén & Muthén, 1998–2017).

Third, in order to test our hypotheses, a structural model was estimated based on the best‐fitting measurement model. Here, the directional relationships between latent factors were estimated through a path model. We simultaneously tested all hypotheses by regressing stress and physical symptoms (Time 2) on anxiety, COVID‐19 risk, family identification, and identification with humankind (all measured at Time 1). Stress and physical symptoms were allowed to covary.

## Results

### Dropout analyses

Participants who only participated in the first survey were significantly younger (*M* = 41.11, *SD* = 15.63) than those who participated in both surveys (*M* = 51.14, *SD* = 13.76), *t* (810.64) = −11.80, *p* < .001. However, there were no gender differences between second and first time only participants (*χ^2^* (1) = 1.37, *p* = .242)^3^.

Regarding the independent variables, there were no differences between second and first time only participants in terms of health‐related anxiety (*t* (1434) = −.563, *p* = .574), family identification (*t* (1434) = −1.51, *p* = .13), and identification with humankind (*t* (1434) = .85, *p* = .393). However, participants who only participated at Time 1 showed higher COVID‐19 risk scores (*M* = .24, *SD* = .24; *t* (775.40) = 3.23, *p* = .001) than participants who participated in both surveys (*M* = .20, *SD* = .20).

### Descriptive statistics and correlations

The descriptive statistics, correlations, and composite reliabilities are summarized in Table 3. All instruments showed acceptable levels of lower (Cronbach Alpha > 0.70) and upper bound limits (Composite reliability > 0.70). The results showed that all scales and subscales were normally distributed with the exclusion of family identification.

**Table 3:** Means (*M*), standard deviations (*SD*), skewness (*SK*), kurtosis (*Rku*), composite reliability (*CR*), and correlations between all variables based on *N* = 974

	*M*	*SD*	*SK*	*Rku*	*CR*	1	2	3	4	5	6	7	8	9
1 Health‐related anxiety	3.38	0.88	‐0.49	0.21	.83									
2 Risk factors	0.20	0.20	0.69	‐0.03	‐	.17***								
3 Family identification	4.52	0.82	‐2.20	5.17	.97	.09**	.11**							
4 Identification with humankind	3.87	0.84	‐0.85	0.91	.90	.19***	.09**	.26***						
5 Stress	2.09	0.59	0.50	‐0.12	.93	.25***	.04	‐.10**	‐.15***					
6 Tension	2.03	0.75	0.63	‐0.10	.87	.22***	.06	‐.09**	‐.10**	.92***				
7 Worries	1.94	0.72	0.73	‐0.07	.87	.24***	.06	‐.11**	‐.13***	.89***	.81***			
8 Joy	2.50	0.65	0.01	‐0.40	.83	‐.16***	.02	.15***	.18***	‐.78***	‐.65***	‐.59***		
9 Demands	1.87	0.66	0.62	‐0.08	.83	.21***	.05	‐.01	‐.10**	.80***	.68***	.62***	‐.43***	
10 Physical Health	2.03	0.96	1.00	0.41	.88	.22***	.13***	‐.04	‐.02	.53***	.55***	.52***	‐.34***	.40***

*p < .05; ** p < .01; ***p < .001.

Mean scores showed that participants were strongly identified with their families, which resulted in a left‐skewed distribution. Compared to family identification, the identification with humankind was lower. Participants reported average levels of anxiety, while the mean risk score was low. Further, the majority of participants found that at least one of the risk factors to be applicable. At Time 2, low to medium stress and physical ill‐health symptoms were reported.

Almost all scales were significantly associated. Based on Cohen (1988), there was a medium positive relation between family identification and identification with humankind. Notably, both identity factors were positively associated with health‐related anxiety and risk (the respective correlational coefficients ranging from .09 to .19 indicate a small relationship between these predictive factors). Health‐related anxiety and risk showed both small positive relations to physical symptoms, whereas only health‐related anxiety but not risk correlated positively with stress at Time 2 (correlation coefficients indicating a medium‐sized effect). Thus, these correlations provide initial support for Hypotheses 2, 3, and 4 as we expect health‐related anxiety and COVID‐19 risk factors to be positively associated with stress and physical symptoms. Finally, family identification and identification with humankind showed small negative correlations with stress at Time 2 but were unrelated to physical symptoms at Time 2, which provides initial support for Hypotheses 5, 6 and 7.

#### Risk factors

In Table 4, the frequency and percentages of the participants’ responses to the five risk statements at Time 1 are presented. Most participants indicated that they did not take care for any individuals being at high risk (73.7%). However, at Time 1, 43.1% lived with or close by a high‐risk individual. Only a small minority specified to have a family member or close friend infected with coronavirus (2.7%). The majority of participants (95.1%) responded that they had not visited a high‐risk area recently. Finally, 10.5% of the respondents indicated to live in a high‐risk area.

**Table 4:** Risk factors: Numbers (n) and percentages (%) based on *N* = 974

	Yes		No	
	*n*	%	*n*	%
Are you taking responsibility for people who are exposed to high risk (e.g., taking care of parents/grandparents)?	256	26.3	718	73.7
Are people who are exposed to high‐risk (elderly, chronically ill) living in your household or close by?	420	43.1	554	56.9
Has a family member or close relative/ friend been diagnosed with coronavirus?	26	2.7	948	97.3
Do you live in a high‐risk area (with many documented cases of coronavirus)?	102	10.5	872	89.5
Have you recently visited a high‐risk area?	48	4.9	926	95.1

### CFA of competing measurement models

Observed measures were treated as indicators for latent factors. One item of the stress‐scale (‘You feel rested’) was removed due to a non‐significant loading in all models. To enhance model fit for the best‐fitting measurement model, we further permitted the residual error terms of item 4 and item 5 of the anxiety scale and item 7 and item 8 from the physical symptom scale to correlate in all models. The risk score was included directly into the five different models (Treiblmaier, Bentler, & Mair, 2011; Kline, 2015). The following CFA models were tested:

- Model 1: A one‐factor model with all items from all instruments loading onto a common first‐order factor.
- Model 2: Family identification, identification with humankind, anxiety, physical health, and stress were each specified as single factor latent variables.
- Model 3: Anxiety and physical health were specified as first‐order latent factors with items loading onto their *a priori* factors. The observed variables for measuring family identification and identification with humankind loaded on a first‐order latent identity factor. Stress was specified as a hierarchical second‐order latent factor, compromising four first‐order latent factors (tension, worries, joy, and demands which were measured by four, five, five and five observed variables, respectively). First‐order factors on stress were constrained to be equal in order to establish convergence. This model was specified to ensure the difference between family identification and identification with humankind.
- Model 4: Family identification, identification with humankind, and physical health were specified as first‐order latent factors with items loading onto their *a priori* factors. The observed variables for measuring anxiety and stress loaded on a first‐order latent stress anxiety factor. This model was specified to ensure the difference between health‐related anxiety and stress.
- Model 5: Family identification, identification with humankind, anxiety, and physical health were specified as first‐order latent factors with items loading onto their *a priori* factors. Stress was specified as a hierarchical second‐order latent factor, comprising out of four first‐order latent factors (tension, worries, joy, and demands).

The model which fitted the following fit indices best was chosen for further analysis: (1) lowest chi‐square‐value, (2) root mean square error of approximation (RMSEA: <0.06), (3) standardized root mean residual (SRMR: <0.08), (4) Tucker‐Lewis Index (TLI: >0.90), (5) comparative fit index (CFI: >0.90) and (6) smaller AIC and BIC values (Wang & Wang, 2012). Measurement quality was a function of significant *a priori* factor loadings (*λ* > .40; *p* < .01), small residual variances centred around zero, and the absence of multicollinearity between factors (Asparouhov & Muthén, 2009). The Sattora‐Bentler scaled chi‐square different test was used to determine the differences between competing measurement models (Satorra & Bentler, 2010).

#### CFA results

The model fit statistics for the five models are summarized in Table 5 and were compared through the Satorra and Bentler (2010) scaled chi‐square correction method. The results showed that Model 5 (χ^2^_(764, N=974_) = 2404.33; *p *< .001, *scaling correction factor for MLR* = 1.15, CFI = 0.92; TLI = 0.92; RMSEA = 0.047 [CI: 0.045, 0.049]; SRMR = 0.05) fitted the data better than Model 1 (Sartorra‐Bentler scaled Δχ^2^ = 5947.39, Δ *df* = 14, Δ *test scaling correction* = 2.82, *p* < .001) and Model 2 (Sartorra‐Bentler scaled Δχ^2^ = 369.70, Δ *df* = 4, Δ *test scaling correction* = 3.07, *p* < .001), indicating the existence of the four stress subscales tension, worries, joy, and demands and their loading on an overall second‐order stress factor. Additionally, according to the fit indices, Model 5 was superior to Model 3 (Sartorra‐Bentler scaled Δχ^2^ = 1082.59, Δ *df* = 7, Δ *test scaling correction* = 2.25, *p* < .001) and Model 4 (Sartorra‐Bentler scaled Δχ^2^ = 1238.19, Δ *df* = 8, Δ *test scaling correction* = 2.12, *p* < .001), supporting the differentiation between family identification and identification with humankind and stress and health‐related anxiety, respectively. Model 5 also showed acceptable levels of measurement quality with all factor loadings exceeding the suggested thresholds (*λ* > 0.40; *p* < 0.01), standardized residual variances ranging from .00 to .77, and no indication of multicollinearity between latent factors. Model 5 was therefore retained for further analyses.

**Table 5:** Fit indices of the five competing measurement models based on *N* = 974

Model	χ^2^	*df*	*c*	*p* value	CFI	TLI	RMSEA	SRMR	95% C.I		AIC	BIC
									RMSEA			
									LL	UL		
Model 1	16542.06	778	1.18	0.00	0.26	0.22	0.144	0.26	0.142	0.146	101230.72	101836.02
Model 2	3362.04	768	1.16	0.00	0.88	0.87	0.059	0.06	0.057	0.061	85629.65	86283.76
Model 3	4484.79	771	1.16	0.00	0.83	0.82	0.070	0.08	0.068	0.072	86936.43	87575.89
Model 4	4641.17	772	1.16	0.00	0.82	0.81	0.072	0.08	0.070	0.074	87112.15	87746.73
Model 5	2404.33	764	1.15	0.00	0.92	0.92	0.047	0.05	0.045	0.049	84523.15	85196.79

χ^2^ = Chi‐square; *df* = degrees of freedom; *c* = scaling correction factor; TLI = Tucker‐Lewis Index; CFI = Comparative Fit Index; RMSEA = Root Mean Square Error of Approximation; SRMR = Standardized Root Mean Square Residual; AIC = Akaike Information Criterion; BIC = Bayes Information Criterion; LL = Lower Level; UL = Upper Level.

#### Structural models and hypotheses testing

**Figure 2:**
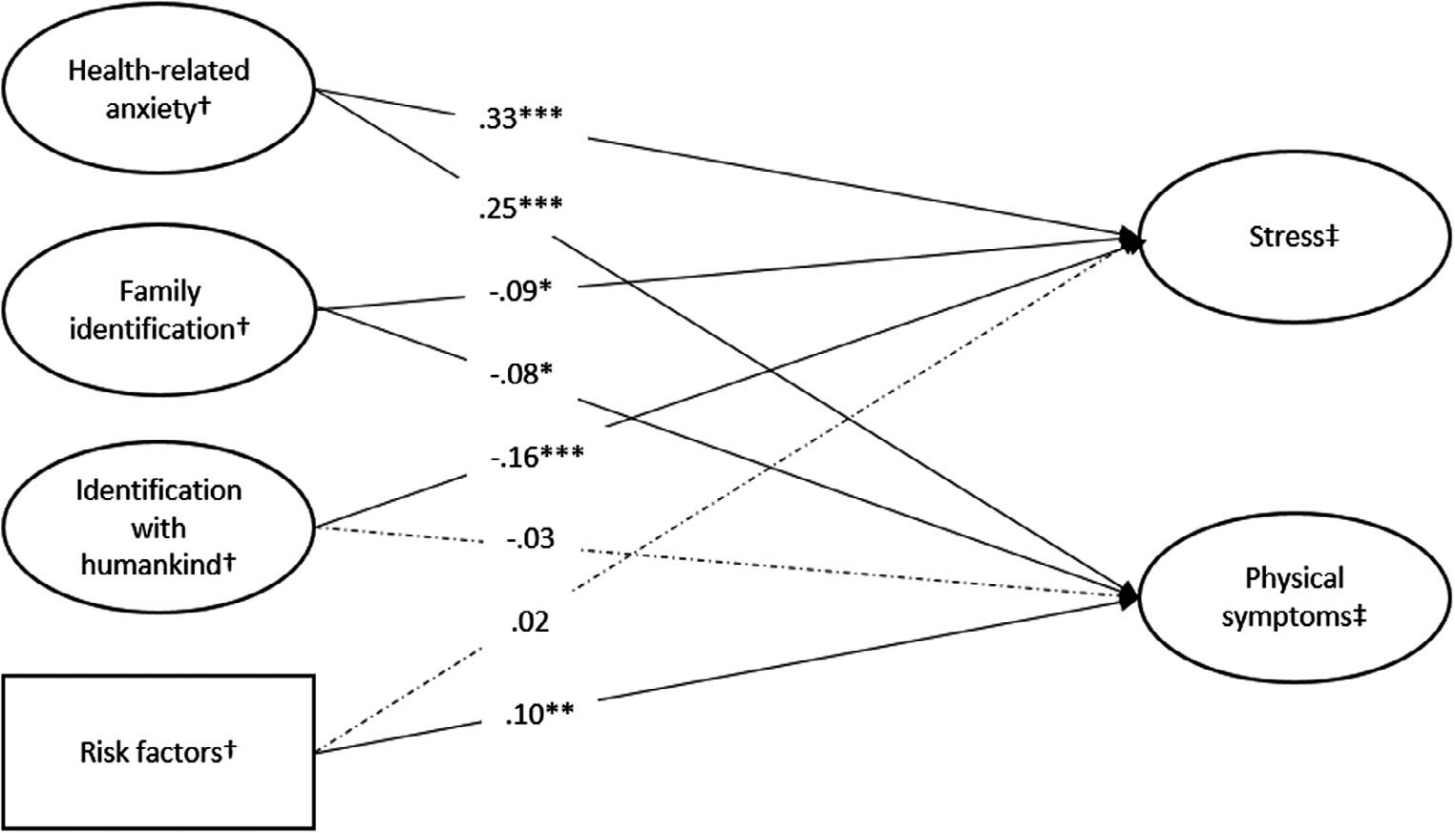
Standardized results of the structural model. Rectangles are observed variables, circles are latent variables; single‐headed arrows represent regression paths with standardized regression coefficient; dashed lines indicate non‐significant relations between variables. *p < .05; **p < .01; ***p < .001; ^†^ measured at Time 1. ^‡^ measured at Time 2.

The structural path model was estimated based on the best‐fitting measurement model (Model 5; see also Figure 2). This model showed good fit (χ^2^_(765,_ *_N_*_=974)_ = 2468.55, *scaling correction factor for MLR* = 1.15, RMSEA = .05 [95% CI: 0.046, 0.050], CFI = .92, TLI = .91, SRMR = .06). H_1_ proposed that risk at Time 1 will be positively associated with stress at Time 2 and H_2_ proposed that risk will be positively associated with physical symptoms at Time 2. Not supporting H1, we did not find a relation between risk and stress (γ = .02, *SE* = .03, *p* = .459, 95% CI: −0.038, 0.083). Supporting H_2,_ we found a positive association between risk and physical symptoms (γ = .10, *SE* = .03, *p* = .004, 95% CI: 0.032, 0.166). H_3_ and H_4_ predicted that health‐related anxiety at Time 1 will be positively related to stress and physical symptoms at Time 2 respectively. Supporting H_3_ and H_4_, anxiety was positively associated with stress (γ = .33, *SE* = .03, *p* < .001, 95% CI: 0.264, 0.398) and physical symptoms (γ = .25, *SE* = .03, *p* < .001, 95% CI: 0.184, 0.316) at Time 2. H_5_ proposed a negative relation between family identification at Time 1 and stress at Time 2 and H_6_ proposed a negative relation between family identification at Time 1 and physical symptoms at Time 2. Our results support H_5_ and H_6_ as family identification was negatively related to stress (γ = −.09, *SE* = .04, *p* = .017, 95% CI: −0.161, −0.016) and physical symptoms (γ = −.08, *SE* = .04, *p* = .022, 95% CI: −0.156, −0.012). Finally, we predicted that identification with humankind at Time 1 would be negatively related with stress (H_7_) and physical symptoms (H_8_) at that Time 2. Supporting H_7_, but not H_8_, identification with humankind was negatively associated with stress (γ = −.16, *SE* = .04, *p* < .001, 95% CI: −0.233, −0.078) but not with physical symptoms (γ = −.03, *SE* = .04, *p* = .448, CI: −0.101, 0.045).

As younger people dropped out of the study and did not participate at Time 2, we also run the analysis controlling for participants' age. Model fit was good (χ^2^_(804,_ *_N_*_=974)_ = 2708.23, *scaling correction factor for MLR* = 1.15, RMSEA = .05 [95% CI: 0.047, 0.051], CFI = .91, TLI = .91, SRMR = .07). Age was negatively associated with stress (γ = −.25, *SE* = .03, *p* < .001, 95% CI: −0.313, −0.193) and physical symptoms (γ = −.15, *SE* = .03, *p* < .001, 95% CI: −0.213, −0.080). However, including age as a covariable did not change our results.

## Discussion

The purpose of this study was to investigate how contextual risk factors, health‐related anxiety, as well as identification with one´s family and with humankind, relate to stress and physical symptoms during the COVID‐19 pandemic. We found partial support for our predictions as health‐related anxiety was positively associated with stress and physical symptoms, whereas risk factors were only related to physical symptoms. Furthermore, both family identification and identification with humankind were negatively associated with stress. However, only family identification but not identification with humankind was negatively related to physical symptoms.

In line with Hypothesis 2 but contrary to Hypothesis 1, COVID‐19 risk factors were only positively related to physical symptoms, but not to stress. One explanation for this missing relation could be that only a minority of participants indicated that they lived in high‐risk areas or experienced a person close to them being infected with COVID‐19 (when the survey was conducted). Although relatively more participants cared for or lived with a member of a risk group, it could be that they did not necessarily perceive this as a problem or burden. In fact, the German government advised people who were at high risk to restrict their activities even more (compared to the rest of the population) to further decrease their risk of infection. Thus, the desired support provided by ‘pandemic caregivers’ might only include minor activities, like grocery shopping or more frequent phone calls, which might not necessarily increase stress perceptions. Taken together, the COVID‐19 risk factors per se are not stress‐inducing as stress responses depend rather on the subjective interpretation of one’s own situation (i.e., health‐related anxiety). Indeed, our result shows that health‐related anxiety was positively related to stress, which is in line with our hypothesis. By embedding these results into the transactional stress model (Lazarus & Folkman, 1984), health‐related anxiety is in line with the primary appraisal process. During this process, people evaluate to what extend the coronavirus poses a threat to themselves and their social groups. This implies that re‐evaluating the coronavirus as less dangerous for one’s own health and the health of other people could reduce stress responses.

Furthermore, health‐related anxiety was also positively associated with physical symptoms, which is in line with previous findings (Suinn, 2001). These results also mirror concerns that the anxiety due to the COVID‐19 pandemic can lead to declining physical health – even without an actual COVID‐19 infection (Heisz, 2020, March 22). This is because the sole expectation of an aversive event activates neurological fear responses, keeping our body in a constant alert (Phelps et al., 2001). Thus, anticipating an aversive event like a COVID‐19 infection over a more extended period can lead to chronic stress exposure which impairs the immune system and, in turn, increases the vulnerability to physical ill‐health conditions (Cohen et al., 2012; for a meta‐analysis on chronic stress exposure and the effects on the humane immune system see Segerstrom & Miller, 2004). Our results imply that health‐related anxiety transfers to other social groups of an individual, which can elicit mental and physical ill‐health symptoms. Conclusively, identifying with different social groups is health beneficial, but it also provides a foundation for feeling anxious and concerned about one’s own social groups.

### Social cure despite social restrictions

Family identification was negatively related to stress and physical symptoms. This result is in line with research on the social cure phenomenon, which shows that group identification positively affects psychological *and* physical health (Jetten et al., 2012). This health beneficial effect of family identification may partially be attributed to the availability of social resources and mutual support within the family, but might also be due to increased stress‐related self‐efficacy or locus of control (Greenaway et al., 2015; Junker et al., 2019; Khan et al., 2014). Importantly, contextual factors (e.g., living alone, being hospitalized, or financial deprivation) might weaken or even invert the curing effect of family identification into a social curse. For instance, family identification might be associated with more – rather than less – strain for hospitalized individuals or those in special care units. As these were not permitted to see their families, they might particularly suffer under the separation (Hart, Turnbull, Oppenheim, & Courtright, 2020; Luttik et al., 2020). On the contrary, living together as a family during the COVID‐19 pandemic is not beneficial per se, as some family members also perceived it as rather burdensome (Evans, Mikocka‐Walus, et al., 2020). Additionally, strict lockdown measures and rare opportunities to leave one’s home raised concerns about increasing (unreported) cases of domestic violence (Evans, Lindauer, Lindauer, & Farrell, 2020). Conclusively, future research should take into account living situations and relationships between household members.

While identification with humankind was negatively related to stress, there was no association with physical symptoms. The former result highlights the importance of symbolic group memberships for the individuals’ mental health. Similarly, Khan et al., (2020) reported the health beneficial effects of national identification and concluded that ‘identification with large‐scale groups arguably provides stable anchors in an otherwise rapidly changing world’ (p. 209). As the COVID‐19 pandemic is an international phenomenon characterized by rapid changes and unknown progression (e.g., steady rise in case numbers, constant adaptions to governmental restrictions), individuals may particularly seek psychological security and stabilization. Feeling a sense of ‘we‐ness’ and knowing that people worldwide are going through the same stressful experience may provide such a secure environment during these turbulent times (Dovidio, Ikizer, Kunst, & Levy, 2020; Gloster et al., 2020; Van Zyl, 2021; van Zyl, Rothmann, & Zondervan‐Zwijnenburg, 2021). Through the feeling of being in this pandemic together, people may experience this low‐control situation as more controllable. Indeed, the results of an experimental study by Greenaway et al., (2015) support this assumption as they showed that national identification has a positive impact on control perception in a low‐control situation which prevented a decrease in well‐being. Alike identification with humankind, national identification consists of a psychological component and the impossibility to interact with every group member directly. During times of uncertainty, identification with humankind might serve to maintain the perception of control and is therefore positively related to individual mental health.

The fact that we could not find the proposed association between identification with humankind and physical symptoms might be due to the operationalization of physical health. In the present study, we measured the frequency of objective physical symptoms, whereas in studies that reported relations of identification with large‐scale groups and physical health, and assessed the perceived physical health (Khan et al., 2020; Ysseldyk, Haslam, & Haslam, 2013). Hence, especially the identification with a larger group such as humankind might be related to the negative appraisal of physical health but not the occurrence of physical symptoms per se.

Furthermore, individuals who experience physical symptoms might benefit more from direct support in form of distraction and encouragement which can be provided by proximal (e.g., family) but not by larger and more distant groups (e.g., humanity). Concerning this matter, we are aware that we chose two extreme social groups on the physical distance spectrum. Other critical groups worth studying are communities or neighbourhoods, because they represent some kind of middle ground between family and humankind, and furthermore, community identification improves well‐being (McNamara et al., 2021). In fact, as a response to the COVID‐19 pandemic, community aid groups have been formed worldwide, which enabled mutual support (McDermott, 2020, March 27).

### Study limitations

Despite its strength which includes the large and representative sample and the lagged data collection during a time of a major health crisis, our study is not without limitations. Some study‐relevant scales, like health‐related anxiety and risk factors, were formulated specifically to fit this study’s needs and were therefore not validated beforehand. Additionally, the health‐relevant constructs were only measured at Time 2, which does not allow for causal conclusions on the obtained associations to be drawn. Moreover, all study variables were self‐reported, eliciting the risk of a common‐method bias (Podsakoff, MacKenzie, Lee, & Podsakoff, 2003). However, the CFA results support the assumption that this was not the case in the present data as the intended model fit the data better than a one‐factor model. Nevertheless, future studies should attempt to combine self‐report data with objective data. Because of the differences found in the present study compared to previous studies (Khan et al., 2020; Wakefield et al., 2016; Ysseldyk et al., 2013), such a combination may be particularly fruitful for examinations of physical symptoms.

### Conclusion

This study contributes to the existing literature by providing essential insights on the relationship between social identification and mental and physical health during the COVID‐19 pandemic. Results reveal that the subjective threat evaluation of oneself and one’s own social groups showed a stronger negative association with psychological health than the rather objective COVID‐19 risk factors. Beyond this, identifying with one’s family and humankind was beneficial for psychological health by being associated with less stress. Nevertheless, there was evidence that only family identification but not identification with humankind was associated with the occurrence of physical symptoms. These findings suggest that (a) identification with family and identification with humankind are independent factors associated with better mental health, (b) identifying with a proximal group (e.g., family) is more helpful for the individual when dealing with concrete physical symptoms, and (c) the importance of identifying with a large collective in the presence of a worldwide stressor should not be underestimated.

Thus, rising awareness that *everyone* struggles with similar negative experiences due to the COVID‐19 pandemic and the need to combat this threat *collaboratively*, strengthens the perceived identification between people and builds a safer and more secure environment in these challenging times.

## Conflict of interests

The authors declare no conflict of interest.

## Author contributions

Svenja Frenzel was involved in writing the original draft and performing formal analysis. Svenja Frenzel, Nina M. Junker, Jan A. Häusser, Andreas Mojzisch, Valerie A. Schury und Rolf van Dick were involved in the conceptualization, methodology, and data curation. Nina M. Junker, Jan A. Häusser und Rolf van Dick were involved in funding acquisition and reviewed and edited the manuscript. Llewellyn van Zyl was involved in formal analysis. Lorenzo Avanzi, Aidos Bolatov, S. Alexander Haslam, Ronit Kark, Ines Meyer, Lucas Monzani, Stephen Reicher, Adil Samekin, Niklas K. Steffens, Liliya Sultanova, and Dina Van Dijk were involved in reviewing the manuscript and data curation.

## Data Availability

The data that support the findings of this study are available from the corresponding author upon reasonable request.

## Footnotes

^1^
One participant did not answer this question.

^2^
As this study was part of a larger research project, further measures were included in the study. Only measures relevant to this paper are reported in this manuscript in more detail. Besides the measures reported here, we assessed identification with several social groups (e.g. family, country, humankind), health‐related anxiety, threat perceptions, adherence to health recommendations, attitudes towards physical distance and panic buying, and COVID‐19 risk factors at Time 1. In addition to the constructs measured at Time 1, we included scales for measuring stress and physical symptoms at Time 2.

^3^
Based on *n* = 461 at Time 1 (first time only responders) and *n* = 974 at Time 2 (participated at Time 1 and Time 2) as one person indicated gender = diverse and was excluded from the analysis.

## Anxiety

- At the moment because of the coronavirus situation, I am feeling anxious about getting seriously ill myself.
- At the moment because of the coronavirus situation, I am feeling anxious about my family/ close friends getting seriously ill.
- At the moment because of the coronavirus situation, I am feeling anxious about my neighbours getting seriously ill
- At the moment because of the coronavirus situation, I am feeling anxious about people in my country getting seriously ill.
- At the moment because of the coronavirus situation, I am feeling anxious about all humans getting seriously ill.

## Risk factors

- Are you taking responsibility for people who are exposed to high risk (e.g., taking care of parents/grandparents)? (*MW*=20.90; 20%)
- Are people who are exposed to high‐risk (elderly, chronically ill) living in your household or close by? (*MW*=27.72; 28%)
- Has a family member or close relative/ friend been diagnosed with coronavirus? (*MW*=27.72; 28%)
- Do you live in a high‐risk area (with many documented cases of coronavirus)? (*MW=14.09;* 14%)
- Have you recently visited a high‐risk area? (*MW*=9.55; 10%)

## Family identification

- I identify with my family.
- I am a part of my family.
- I feel strong ties with my family.
- I am glad to be a part of my family.

## Identification with Humankind

- I identify with other human beings.
- I am a part of humankind.
- I feel strong ties with humankind.
- I am glad to be part of humankind

## Stress

- You feel rested.
- You feel that too many demands are being made on you.
- You have too many things to do.
- You feel you’re doing things you really like.
- You fear you may not manage to attain your goals.
- You feel calm.
- You feel frustrated.
- You are full of energy.
- You feel tense.
- Your problems seem to be piling up.
- You feel you’re in a hurry.
- You feel safe and protected.
- You have many worries.
- You enjoy yourself.
- You are afraid for the future.
- You are light‐hearted.
- You feel mentally exhausted.
- You have trouble relaxing.
- You have enough time for yourself.
- You feel under pressure from deadlines.

## Physical symptoms

- Stomach ache
- Being easily exhausted
- Palpitations or heart pounding
- Dizziness
- Tiredness
- Feeling bloated or distended
- Neck or shoulder pain
- Backache
